# Beginning Kidney Replacement Therapy Before or After Liver Transplantation: Does the Timing of Dialysis Matter? A Cohort Study

**DOI:** 10.1155/joot/6880648

**Published:** 2026-10-19

**Authors:** Patricia Faria Scherer, Adriano Luiz Ammirati, Bento Fortunato Cardoso dos Santos, Julio Cesar Martins Monte, Marcelo Costa Batista, Marisa Petrucelli Doher, Oscar Fernando Pavão dos Santos, Thais Nemoto Matsui, Thiago Domingos Corrêa, Virgílio Gonçalves Pereira, Carolina Devite Bittante, Marcelino de Souza Durão

**Affiliations:** ^1^ Nephrology Division Federal University of Sao Paulo (UNIFESP) Sao Paulo Brazil unifesp.br; ^2^ Nephrology Division Hospital Israelita Albert Einstein Sao Paulo Brazil einstein.br; ^3^ Intensive Care Unit Hospital Israelita Albert Einstein Sao Paulo Brazil einstein.br; ^4^ Dialysis Center Hospital Israelita Albert Einstein Sao Paulo Brazil einstein.br; ^5^ Internal Medicine Albert Einstein Israeli Faculty of Health Sciences (FICSAE) Hospital Israelita Albert Einstein Sao Paulo Brazil einstein.br; ^6^ Infectious Diseases Care Team Hospital Israelita Albert Einstein Sao Paulo Brazil einstein.br; ^7^ Kidney Transplant Unit Hospital Israelita Albert Einstein Sao Paulo Brazil einstein.br

**Keywords:** dialysis, graft dysfunction, intensive care unit, kidney replacement therapy, liver transplantation

## Abstract

**Introduction:**

Acute kidney injury (AKI) in liver transplant patients is associated with increased morbidity, mortality, and reduced graft survival. However, the impact of the timing of dialysis initiation—before versus after transplantation—remains unclear.

**Objectives:**

To characterize liver transplant recipients with Kidney Disease: Improving Global Outcomes (KDIGO) Stage 3 AKI–requiring dialysis (AKI‐D) according to the timing of kidney replacement therapy (KRT) initiation (pre‐ versus posttransplant) and to evaluate 90‐day mortality. Secondary objectives included identifying independent predictors of dialysis dependence and assessing liver graft function at 90 days.

**Methods:**

This retrospective, single‐center cohort study included all consecutive adult liver transplant recipients who received dialysis in a Brazilian intensive care unit (ICU) between 1999 and 2015. The study was approved by the Research Ethics Committee of the Federal University of Sao Paulo (UNIFESP), with a waiver of informed consent. Patients were grouped according to the timing of dialysis initiation: pre‐ or postliver transplantation. Multiple logistic regression was used to identify predictors of 90‐day mortality and dialysis dependence. Ninety‐day liver graft survival was analyzed using a multivariate Cox model.

**Results:**

A total of 356 liver transplant patients with AKI‐D were included. Of these, 109 (30.6%) initiated dialysis within 4 weeks before transplantation and 247 (69.4%) within 4 weeks after transplantation. The timing of dialysis initiation was not associated with 90‐day mortality or dialysis dependence. Independent predictors of 90‐day mortality included graft dysfunction and use of continuous KRT as the initial modality. Impaired pretransplant renal function and a history of diabetes mellitus were predictors of 90‐day dialysis dependence. Posttransplant dialysis was associated with a twofold increased risk of liver graft failure.

**Conclusion:**

The timing of dialysis initiation (pre‐ versus posttransplantation) was not associated with 90‐day mortality or dialysis dependence. However, initiation of dialysis after transplantation was associated with an increased risk of liver graft dysfunction.

## 1. Introduction

Liver transplantation has become the standard of care for patients with life‐threatening liver disease [1]. Liver disease induces organ crosstalk (pathophysiological communication between organs), affecting the kidneys, cardiovascular system, nervous system, and gastrointestinal tract [2, 3]. The hepatorenal connection is characterized by complex hemodynamic and inflammatory mechanisms that work synergistically to cause acute kidney injury (AKI) [3]. The pathogenesis of AKI in liver disease is frequently multifactorial [4]. AKI is a common complication among liver transplant recipients in both the pre‐ and posttransplant periods [5, 6] and represents a significant risk factor for morbidity and mortality [3, 7].

In the pretransplantation phase, AKI represents a marker of the severity of liver disease [8]. Hypovolemia, intrinsic renal lesions, and hepatorenal syndrome (HRS) are common causes of AKI in cirrhotic patients [3]. Systemic vasodilatation, hypoperfusion, endotoxinemia, cardiac dysfunction, and direct nephrotoxicity are relevant mechanisms that predispose these patients to kidney injury [4, 7]. In acute‐on‐chronic liver failure (ACLF), intense systemic inflammation and oxidative stress play a role in multiple organ failure, including kidney injury [9]. Pathogen‐associated molecular patterns (PAMPs) and damage‐associated molecular patterns (DAMPs) released during liver damage and endotoxemia interact with receptors on renal tubular epithelial cells, such as Toll‐like receptor 4. This cascade amplifies the inflammatory response and drives tubular and microcirculatory dysfunction, culminating in AKI [4]. AKI is a significant concern in the management of liver transplant candidates, as serum creatinine is a critical component of the Model for End‐Stage Liver Disease (MELD) scoring system [10]. Kidney replacement therapy (KRT) is often used as a bridge to transplantation in patients with liver failure [8]. The proportion of patients on dialysis prior to liver transplantation increased by 41.3% from the pre‐MELD to the post‐MELD era [11]. The severity of pretransplant kidney injury increases the risk of prolonged kidney dysfunction after liver transplantation [12].

In the posttransplantation phase, the incidence of AKI is a dynamic phenomenon reflecting diverse pathophysiological processes [13]. The etiology of AKI among liver transplant recipients involves preoperative renal reserve, ischemic injury, hemodynamic instability, inflammation, and drug‐induced nephrotoxicity [14–16]. Furthermore, several factors have been implicated in the development of AKI after liver transplantation: preexisting renal disease [16–19], severity of underlying liver disease [16, 19], female sex and diabetes mellitus [14], overweight and obesity [16, 18–20], and preoperative infection [18, 19]; donor and graft characteristics (donor age, donor type, graft steatosis [18], and cold and warm ischemia times [19]); surgical procedure and perioperative management variables (surgical technique [18], postreperfusion syndrome [19–21], volume of blood component transfusion [16–20], infectious complications [16, 18–20], and immunosuppression regimens [16, 18, 20]). The incidence of renal dysfunction following liver transplantation exceeds 50% [14, 17, 22–26], with 7.7%–22.5% of patients requiring KRT [17, 19, 22, 24–27]. Postliver transplant AKI is also a significant risk factor for both chronic kidney disease (CKD) and liver graft dysfunction [14, 20, 23–26, 28]. The coexistence of early allograft dysfunction and AKI exerts a synergistic negative impact on patient and liver graft survival [29].

AKI in liver transplant recipients has both short‐ and long‐term consequences, including increased mortality [23, 24, 28, 30], progression to CKD [14, 30], and reduced liver graft survival [14, 23]. Although successful liver transplantation may reverse the course of liver disease and contribute to renal recovery, the impact of the timing of dialysis initiation on clinical outcomes remains unclear. Given the exploratory nature, this study aimed to evaluate the effect of the timing of KRT initiation (pre‐ versus postliver transplantation) on 90‐day mortality, dialysis dependence, and graft survival in liver transplant recipients who developed dialysis‐requiring AKI (AKI‐D).

## 2. Methods

### 2.1. Study Design

This was a retrospective, single‐center cohort study based on a review of medical records. The study protocol was approved by the Research Ethics Committee of the Federal University of Sao Paulo (UNIFESP) under CAAE No. 27698819300005505, with a waiver of informed consent. The study is reported in accordance with the Strengthening the Reporting of Observational Studies in Epidemiology (STROBE) statement [31].

### 2.2. Setting

The study was conducted in the Adult Intensive Care Unit (ICU) of Hospital Israelita Albert Einstein, a private quaternary care hospital located in Sao Paulo, SP, Brazil.

### 2.3. Study Participants

All consecutive patients who underwent liver transplantation and received KRT in the ICU between January 1, 1999, and December 31, 2015, were considered eligible. A convenience sample was therefore used, and no a priori sample size calculation was performed. We included liver transplant recipients aged 18 years or older who initiated KRT in either the pre‐ or posttransplant period. For inclusion in this study, KRT initiation had to occur within a 4‐week window pre‐ and postliver transplantation, as the renal recovery rate declines significantly beyond this period and to avoid overlap with the simultaneous liver–kidney allocation policy [32].

Patients who initiated KRT outside the 4‐week window relative to the liver transplant date were excluded. We also excluded individuals with dialysis‐dependent CKD, those who underwent simultaneous liver–kidney transplantation, or those who had received a kidney transplant prior to liver transplantation.

Following the study period, the institutional organ transplant program, including liver transplant candidates, began operating in another hospital within the Brazilian public health system (Sistema Único de Saúde, SUS) network, a relocation that persisted for the subsequent 6 years. During this period, pre‐, peri‐, and posttransplant care was provided by the multidisciplinary team affiliated with the Hospital Municipal Gilson de Cassia Marques de Carvalho, precluding the inclusion of patients transplanted after 2015.

### 2.4. Primary and Secondary Outcomes and Other Variables

Our primary outcome of interest was 90‐day mortality. Secondary outcomes included 90‐day dialysis dependence and graft survival in liver transplant recipients who developed an episode of AKI‐D.

Collected variables included demographic characteristics, comorbidities, presence of sepsis [33], baseline renal function parameters (serum creatinine and estimated glomerular filtration rate [34–37]), etiology of AKI, and liver disease–related information (cause of liver disease and MELD score [38]). We also collected KRT‐related variables (initial dialysis modality and duration of treatment) and transplant‐specific characteristics (donor type and need for retransplant). In addition, data on ICU and hospital length of stay (LOS) and 90‐day mortality were recorded.

### 2.5. Data Collection

Data were obtained from the Epimed Monitor System (Epimed Solutions, Rio de Janeiro, Brazil), an electronic structured case report form in which trained ICU case managers prospectively entered patient data [39], as well as from a nephrology‐specific database developed by the hospital’s multidisciplinary team. All data were anonymized prior to analysis.

### 2.6. Renal Injury Definitions and KRT Modalities

AKI was defined according to the Kidney Disease: Improving Global Outcomes (KDIGO) criteria [40]. Baseline renal function was defined using the most recent serum creatinine values available prior to hospitalization [34–36]. When serum creatinine values were unavailable, the first creatinine measurement upon admission was used [35, 41]. Baseline glomerular filtration rate was estimated using the CKD Epidemiology Collaboration (CKD‐EPI) 2021 equation (37). Recovery of renal function was defined as independence from KRT [36, 42].

Traditional indications for KRT in the institution were uremia, fluid overload, refractory hyperkalemia, and metabolic acidosis [43]. Nonurgent indications depended on the patient’s clinical condition and the nephrologist’s judgment. The KRT modalities used were intermittent hemodialysis (IHD) and continuous venovenous hemodiafiltration (CVVHDF). CVVHDF was used in patients with hemodynamic instability, severe neurological injury, and/or intracranial hypertension, requiring strict metabolic and volume control. The Prisma or Prismaflex (Gambro Lundia AB, Lund, Sweden) systems were used with M100 or M150 filters (AN69 dialyzer membrane) and a blood flow rate of 100–120 mL/min. Since 1999, regional citrate anticoagulation has been employed [44], with citrate infusion limited to 2.5 mmol/L in patients with liver dysfunction (INR ≥ 2.5). The minimum prescribed continuous dialysis dose was 25 mL/kg/h [40]. For IHD, the Fresenius 4008 hemodialysis machine (Fresenius Medical Care AG, Bad Homburg, Germany) was used, with a blood flow rate of 300–400 mL/min and dialysate flow rate of 500–800 mL/min. When feasible, the dialysis target was a urea reduction ratio ≥ 65% and Kt/V ≥ 1.2 per session [40]. Anticoagulation was not routinely used during IHD; instead, priming with 100 mL of 0.9% NaCl every 15 min was performed.

### 2.7. Statistical Analysis

Categorical variables are expressed as absolute and relative frequencies, while continuous variables are reported as medians and interquartile ranges (IQRs). Associations between categorical variables and the timing of dialysis initiation (pre‐ vs. postliver transplantation) were assessed using the chi‐square test or Fisher’s exact test, as appropriate. For continuous variables, Student’s *t*‐test for independent samples or the nonparametric Mann–Whitney *U* test was used, depending on data distribution. Normality was evaluated using the Shapiro–Wilk test.

Independent predictors of 90‐day mortality and dialysis dependence at 90 days after liver transplantation were identified using multivariable analysis. Variables with a *p* value < 0.20 in the univariate analysis were included in a multiple logistic regression model, from which odds ratios (ORs) and corresponding 95% confidence intervals (CIs) were estimated. A stepwise selection method was applied to build the final model. Model calibration was assessed using the Hosmer–Lemeshow goodness‐of‐fit test. Multicollinearity among predictor variables was evaluated using the variance inflation factor (VIF), with values greater than five indicating substantial collinearity. The assumption of linearity for continuous predictors was examined both graphically and using the likelihood ratio test.

In the graft survival analysis, liver retransplantation was defined as the event of interest, and the liver graft was used as the unit of analysis. Graft survival at 90 days was estimated using the Kaplan–Meier method. Univariate Cox proportional hazards models identified risk factors for graft failure, with variables reaching a *p* value < 0.20 entering a stepwise multivariate analysis. The proportional hazards assumption was verified via Schoenfeld residuals. To address baseline differences between the pre‐ and posttransplant dialysis groups, we constructed two separate multivariable models; this prevented multicollinearity between estimated GFR and MELD scores, both of which derive from creatinine‐based equations. These models were adjusted for recipient age, initial dialysis modality, donor type, sepsis, timing of dialysis initiation, MELD score, and baseline GFR. The first model incorporated the estimated GFR, while the second model evaluated the MELD score.

A *p* value < 0.05 was considered statistically significant. All analyses were performed using *R* software, Version 4.3.4 (https://www.r-project.org).

## 3. Results

### 3.1. Overall Cohort

Between January 1, 1999, and December 31, 2015, a total of 484 patients underwent liver transplantation and received KRT in the ICU. Of these, 42 patients were excluded due to end‐stage kidney disease, liver–kidney transplantation, or previous kidney transplantation. An additional 82 patients were excluded for initiating dialysis outside the 4‐week window prior to or following liver transplantation, resulting in a final cohort of 356 patients. Among the 356 patients included in the analysis, 109 initiated dialysis pretransplantation and 247 posttransplantation (Figure 1).

**FIGURE 1:**
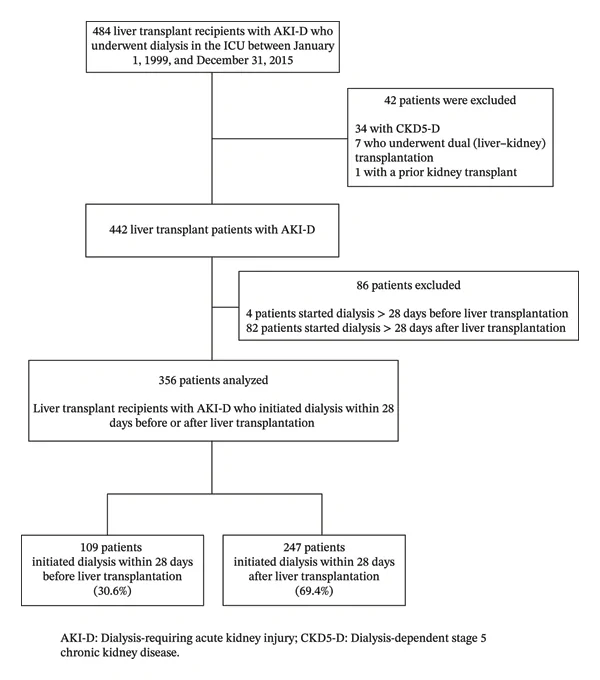
Study flowchart.

The median age of the overall cohort was 53 (42–61) years, and 58.4% were male. Systemic arterial hypertension was present in 7.9% of patients, 4.5% had diabetes mellitus, and 1.4% had heart disease. The baseline creatinine was 0.8 (0.7–1.0) mg/dL. HRS was the primary etiology of AKI, accounting for one‐third of the evaluated cases (33.7%). Viral hepatitis was the main cause of liver disease (40.4%). The mean MELD score was 30 (10.1), and the median BMI was 26.5 (23.6–30.8). Approximately one‐fifth of the patients underwent transplantation prior to the national implementation of the MELD score–based graft allocation (20.8%). The remaining 79.2% underwent transplantation in the post‐MELD era. Most patients received a deceased‐donor graft (91.6%). Sepsis was observed in 16% of cases. Tacrolimus was the preferred calcineurin inhibitor and was used in more than 80% of patients (84.6%). Furthermore, 64% of the patients initiated KRT with CVVHDF. The median duration of dialysis was 16 [8–30] days. The median LOS in the ICU and hospital was 11 [6–20] and 30 [18–48] days, respectively. Overall mortality was 28.4%, and 11.4% of patients remained dialysis‐dependent at 90 days.

Patients who underwent pretransplant dialysis were more frequently diagnosed with severe acute liver failure (SALF) compared to those who initiated dialysis after transplantation (23.8% vs. 7.7%; *p* < 0.001) and had a higher prevalence of sepsis (33.0% versus 8.5%; *p* < 0.001). In contrast, patients who received dialysis posttransplant were older (54 [46–62] versus 49 [36–58] years; *p* = 0.0029), more commonly developed graft dysfunction (39.3% versus 19.3%; *p* < 0.001), and were more likely to undergo retransplantation (30.8% versus 11.9%; *p* < 0.001) (Table 1). The etiology of AKI was not associated with the timing of dialysis. Continuous KRT was the most frequently used initial dialysis modality, particularly among patients who received pretransplant dialysis (71.5% versus 60.7%; *p* = 0.0497). There were no statistically significant differences between the pre‐ and posttransplant groups in total dialysis duration or ICU and hospital LOS (Table 1).

**TABLE 1:** Characteristics of liver transplant recipients undergoing dialysis therapy according to the timing of dialysis initiation.

Characteristics	All patients 356/356 (100%)	Pretransplant dialysis initiation 109/356 (30.6%)	Posttransplant dialysis initiation 247/356 (69.4%)	*p* value
Age, years (median, IQR)	53 (42–61)	49 (36–58)	54 (46–62)	0.0029^†^
Sex (male), *n* (%)	208/356 (58.4)	60/109 (55.0)	148/247 (59.9)	0.390^‡^
Comorbidities, *n* (%)				
Systemic arterial hypertension	28/356 (7.9)	10/109 (9.2)	18/247 (7.3)	0.542^‡^
Diabetes mellitus	16/356 (4.5)	6/109 (5.5)	10/247 (4.0)	0.739^‡^
Heart disease	5/356 (1.4)	2/109 (1.8)	3/247 (1.2)	1.000^‡^
Baseline creatinine, mg/dL (median, IQR)	0.8 (0.7–1)	0.9 (0.7–1.3)	0.8 (0.7–1)	0.0646^†^
Baseline eGFR, mL/min/1,73 m^2^ (median, IQR)	100.2 (76.3–110)	100.1 (64.1–113.7)	100.2 (85.2–109.4)	0.3639^†^
Etiology of AKI, *n* (%)				0.621^‡^
Hepatorenal syndrome	120/356 (33.7)	36/109 (33.0)	84/247 (34.0)	
Sepsis	95/356 (26.7)	30/109 (27.5)	65/247 (26.3)	
Ischemic	87/356 (24.4)	30/109 (27.5)	57/247 (23.1)	
Nephrotoxicity	51/356 (14.3)	13/109 (12.0)	38/247 (15.4)	
Glomerulonephritis	3/356 (0.9)	0/109	3/247 (1.2)	
Cause of liver disease, *n* (%)				0.0021^‡^
Alcohol‐related	42/356 (11.8)	11/109 (10.1)	31/247 (12.5)	
Autoimmune	18/356 (5.0)	5/109 (4.6)	13/247 (5.3)	
Biliary	17/356 (4.7)	7/109 (6.4)	10/247 (4.0)	
Viral hepatitis	144/356 (40.4)	34/109 (31.2)	110/247 (44.5)	
Hereditary	17/356 (4.8)	4/109 (3.7)	13/247 (5.3)	
SALF	45/356 (12.6)	26/109 (23.8)	19/247 (7.7)	
Other	73/356 (20.5)	22/109 (20.2)	51/247 (20.6)	
BMI (median, IQR)^#^	26.5 (23.6–30.8)	26.5 (23.3–30.7)	26.4 (24.2–30.8)	0.5871^†^
MELD score (median, IQR)^∗∗∗^	30 (24–36)	35 (31.2–40)	27 (20–32.2)	< 0.001^§^
Era, *n* (%)^∗^				
Pre‐MELD	74/356 (20.8)	12/109 (11.0)	62/247 (25.1)	0.0001^‡^
Post‐MELD Period 1	131/356 (36.8)	57/109 (52.3)	74/247 (30.0)	
Post‐MELD Period 2	151/356 (42.4)	40/109 (36.7)	111/247 (44.9)	
Donor type, *n* (%)				
Living	26/356 (7.3)	2/109 (1.8)	24/247 (9.7)	0.0114^‡^
Deceased	326/356 (91.6)	107/109 (98.2)	219/247 (88.7)	
Domino transplant	4/356 (1.1)	0/109 (0)	4/247 (1.6)	
Graft dysfunction, *n* (%)	118/356 (33.1)	21/109 (19.3)	97/247 (39.3)	< 0.001^‡^
Type of dysfunction, *n* (%)				1^‡^
Early	92/356 (25.8)	16/109 (14.7)	76/247 (30.8)	
Late	26/356 (7.3)	5/109 (4.6)	21/247 (8.5)	
Retransplantation, *n* (%)	89/356 (25.0)	13/109 (11.9)	76/247 (30.8)	< 0.001^‡^
SALF, *n* (%)	45/356 (12.6)	26/109 (23.8)	19/247 (7.7)	< 0.001^‡^
Sepsis ^∗∗^, *n* (%)	57/356 (16.0)	36/109 (33.0)	21/247 (8.5)	< 0.001^‡^
Immunosuppressive drug, *n* (%)				
Tacrolimus	301/356 (84.6)	88/109 (80.8)	213/247 (86.2)	0.0012^‡^
Cyclosporine	20/356 (5.6)	2/109 (1.8)	18/247 (7.3)	
Other	35/356 (9.8)	19/109 (17.4)	16/247 (6.5)	
Initial dialysis modality				
CVVHDF	228/356 (64.0)	78/109 (71.5)	150/247 (60.7)	0.0497^‡^
Intermittent HD	128/356 (35.9)	31/109 (28.4)	97/247 (39.3)	
Duration of dialysis, days (median, IQR)	16 (8–30)	18.5 (9–36)	16 (8–28)	0.0605^†^
ICU LOS, days (median, IQR)	11 (6–20)	11 (7–23)	11 (6–18)	0.0762^†^
Hospital LOS, days (median, IQR)	30 (18–48)	31 (18–50)	30 (18–46.5)	0.7552^†^
Hospital mortality, *n* (%)	104/356 (29.2)	32/109 (29.4)	72/247 (29.1)	0.5794^‡^
90‐day mortality, *n* (%)	101/356 (28.4)	31/109 (28.4)	70/247 (28.3)	0.9846^‡^

*Note:* Results presented as median (interquartile range) or n/total (%).

Abbreviaitons: BMI, body mass index = weight in kilograms/height in meters^2^; CVVHDF, continuous venovenous hemodiafiltration; GFR, glomerular filtration rate; HD, hemodialysis; LOS, length of stay; MELD, Model for End‐Stage Liver Disease = 10 × (0.957 × ln (creatinine) + 0.378 × ln (bilirubin) + 1.12 × ln (INR)) + 6.43 (the higher the MELD score, the more severe the liver disease and the greater the probability of death); SALF, severe acute liver failure.

^#^Data available for 256/356 patients (71.9%).

^∗^Era: pre‐MELD (January 1, 1999 to June 30, 2006), post‐MELD Period 1 (July 1, 2006 to December 31, 2010), and post‐MELD Period 2 (January 1, 2011 to December 31, 2015).

^∗∗^Focus of infection: intra‐abdominal (50.9%), respiratory tract (26.3%), skin or soft tissue (5.3%), urinary tract (3.5%), bloodstream (3.5%), and other (10.5%).

^∗∗∗^The information pertaining to the MELD score. Data available for 346/356 patients (97.2%).

^‡^ *p* values calculated using the chi‐square test.

^†^ *p* values calculated using the Mann–Whitney *U* test.

^§^ *p* values calculated using the Student’s *t*‐test.

Among the 118 cases of liver graft dysfunction, the most common causes were primary graft dysfunction (50/118; 42.4%), hepatic artery thrombosis (27/118; 22.9%), rejection (15/118; 12.7%), biliary complications (13/118; 11.0%), and recurrence of the underlying disease (9/118; 7.6%); other causes accounted for 4 cases (3.4%).

### 3.2. Predictors of 90‐Day Mortality Among Liver Transplant Recipients Who Developed an Episode of AKI‐D

Liver graft dysfunction (OR = 4.69; 95% CI: 2.25–10.16; *p* < 0.001) and the use of CVVHDF (OR = 3.99; 95% CI: 2.21–7.61; *p* < 0.001) as the initial dialysis modality were identified as independent predictors of increased 90‐day mortality. In contrast, liver retransplantation (OR = 0.44; 95% CI: 0.19–0.97; *p* = 0.019) was an independent predictor of lower 90‐day mortality (Table 2).

**TABLE 2:** Univariate and multivariate logistic regression analyses of factors associated with 90‐day mortality among liver transplant recipients receiving dialysis before or after transplantation.

Predictors	Survivors 255/356 (71.6%)	Nonsurvivors 101/356 (28.4%)	Univariate analysis	*p* value	Multivariate analysis	*p* value
			OR (95% CI)		OR (95% CI)	
Age, years (median, IQR)	53 (43.5–60)	54 (41–61)	1.00 (0.98–1.02)	0.8578		
Sex (male), *n* (%)	157/255 (61.6)	51/101 (50.5)	0.64 (0.40–1.01)	0.0569		
Comorbidities, *n* (%)						
Systemic arterial hypertension	21/255 (8.2)	7/101 (6.9)	0.83 (0.32–1.93)	0.6766		
Diabetes mellitus	12/255 (4.7)	4/101 (3.9)	0.84 (0.23–2.46)	0.7567		
Heart disease	4/255 (1.6)	1/101 (1.0)	0.63 (0.03–4.31)	0.6651		
Baseline creatinine, mg/dL (median, IQR)	0.8 (0.7–1.1)	0.8 (0.7–1)	1.00 (0.68–1.4)	0.992		
Baseline eGFR,mL/min/1,73 m^2^ (median, IQR)	100.8 (75.7–110.3)	98.3 (79.7–109.3)	1.00 (0.99–1.01)	0.7188		
Etiology of AKI, *n* (%)						
Hepatorenal syndrome	94/255 (36.9)	26/101 (25.8)	Reference	0.0254		
Sepsis	58/255 (22.7)	37/101 (36.6)	2.30 (1.27–4.23)			
Ischemic	66/255 (25.9)	21/101 (20.8)	1.15 (0.59–2.21)			
Nephrotoxicity	34/255 (13.3)	17/101 (16.8)	1.80 (0.86–3.72)			
Glomerulonephritis	3/255 (1.2)	0/101 (0)				
Cause of liver disease, *n* (%)						
Alcohol‐related	34/255 (13.3)	8/101 (7.9)	Reference	0.2803		
Autoimmune	11/255 (4.3)	7/101 (6.9)	2.70 (0.79–9.34)			
Biliary	15/255 (5.9)	2/101 (1.9)	0.57 (0.08–2.6)			
Viral hepatitis	105/255 (41.2)	39/101 (38.6)	1.58 (0.70–3.93)			
Hereditary	11/255 (4.3)	6/101 (5.9)	2.32 (0.64–8.23)			
SALF	31/255 (12.1)	14/101 (13.9)	1.92 (0.72–5.39)			
Other	48/255 (18.8)	25/101 (24.7)	2.21 (0.92–5.78)			
BMI (median, IQR)	26.4 (23.9–30.6)	27 (23.4–31.3)	1.01 (0.96–1.07)	0.6383		
MELD (median, IQR)	30 (24–36)	29 (21.8–36)	0.99 (0.97–1.01)	0.3445		
Era, *n* (%)^∗^						
Pre‐MELD	47/255 (18.4)	27/101 (26.7)	Reference	0.2164		
Post‐MELD Period 1	98/255 (38.5)	33/101 (32.7)	0.58 (0.31–1.08)			
Post‐MELD Period 2	110/255 (43.1)	41/101 (40.6)	0.64 (0.35–1.18)			
Donor type, *n* (%)						
Living	15/255 (5.9)	11/101 (10.9)	Reference	0.039		
Deceased	239/255 (93.7)	87/101 (86.1)	0.50 (0.22–1.15)			
Domino transplant	1/255 (0.4)	3/101 (2.9)	4.09 (0.45–89.08)			
Graft dysfunction, *n* (%)	66/255 (25.9)	52/101 (51.5)	3.04 (1.88–4.93)	< 0.001	4.69 (2.25–10.16)	< 0.001
Type of dysfunction, *n* (%)						
Early	48/255 (18.8)	44/101 (43.6)	Reference	0.1171		
Late	18/255 (7.0)	8/101 (7.9)	0.48 (0.18–1.19)			
Retransplantation, *n* (%)	57/255 (22.3)	32/101 (31.7)	1.61 (0.96–2.68)	0.071	0.44 (0.19–0.97)	0.019
SALF, *n* (%)	31/255 (12.1)	14/101 (13.9)	1.16 (0.58–2.25)	0.76651		
Sepsis, *n* (%)	39/255 (15.3)	18/101 (17.8)	1.20 (0.64–2.19)	0.5611		
Immunosuppressive drug, *n* (%)						
Tacrolimus	243/255 (95.3)	58/101 (57.4)	Reference	< 0.001		
Cyclosporine	12/255 (4.7)	8/101 (7.9)	2.79 (1.05–7.06)			
Other	0/255 (0)	35/101 (34.7)				
Initial dialysis modality, *n* (%)						
CVVHDF	142/255 (55.7)	86/101 (85.1)	Reference	< 0.001	3.99 (2.21–7.61)	< 0.001
Intermittent HD	113/255 (44.3)	15/101 (14.8)	0.22 (0.12–0.39)			
Timing of dialysis, *n* (%)						
Preliver transplant	78/255 (30.6)	31/101 (30.7)	Reference	0.9846		
Postliver transplant	177/255 (69.4)	70/101 (69.3)	0.99 (0.61–1.65)			

*Note:* Results presented as median (interquartile range) or n/total (%). OR, odds ratio; 95% CI, 95% confidence interval; CVVHDF, continuous venovenous hemodiafiltration; HD, hemodialysis. For all variables included in the final multivariate logistic regression model, variance inflation factor (VIF) values were below 2.22, indicating no significant multicollinearity. Model calibration, assessed by the Hosmer–Lemeshow goodness‐of‐fit test, showed good fit (*X* ^2^ = 2.49; *p* = 0.48).

Abbreviations: BMI**,** body mass index; GFR, glomerular filtration rate; MELD, Model for End‐Stage Liver Disease; SALF, severe acute liver failure.

^∗^Era: pre‐MELD (January 1, 1999 to June 30, 2006), post‐MELD Period 1 (July 1, 2006 to December 31, 2010), and post‐MELD Period 2 (January 1, 2011 to December 31, 2015).

### 3.3. Predictors of 90‐Day Dialysis Dependence Among Liver Transplant Recipients Who Developed an Episode of AKI‐D

Pretransplant diabetes mellitus was independently associated with dialysis dependence at 90 days (OR = 5.30; 95% CI: 1.39–18.74; *p* = 0.0055), whereas higher baseline eGFR was independently associated with a lower risk of dialysis dependence at 90 days (OR = 0.98; 95% CI: 0.97–0.99; *p* = 0.0053) (Table 3).

**TABLE 3:** Univariate and multivariate logistic regression analyses of factors associated with 90‐day dialysis dependence among liver transplant recipients receiving dialysis before or after transplantation.

Predictors	Nondialysis dependence 210^#^ (88.6%)	Dialysis dependence 27 (11.4%)	Univariate analysis	*p* value	Multivariate analysis	*p* value
			OR (95% CI)		OR (95% CI)	
Age, years (median, IQR)	52.5 (41–60)	56 (47.5–63.5)	1.04 (1.01–1.08)	0.0388		
Sex (male), *n* (%)	128/210 (60.9)	16/27 (59.2)	0.93 (0.42–2.16)	0.8656		
Comorbidities, *n* (%)						
Systemic arterial hypertension	17/210 (8.1)	4/27 (14.8)	1.97 (0.53–5.90)	0.2813		
Diabetes mellitus	7/210 (3.3)	5/27 (18.5)	6.59 (1.82–22.46)	0.0055	5.30 (1.39–18.74)	0.0055
Heart disease	3/210 (1.4)	1/27 (3.7)	2.65 (0.13–21.60)	0.4447		
Baseline creatinine, mg/dL (median, IQR)	0.8 (0.7–1)	1/27 (0.7–2)	1.98 (1.18–3.38)	0.0106		
Baseline eGFR,mL/min/1,73 m^2^ (median, IQR)	101.6 (82.5–110.8)	83.3 (37.5–106.5)	0.98 (0.97–0.99)	0.0014	0.98 (0.97–0.99)	0.0053
Etiology of AKI, *n* (%)						
Hepatorenal syndrome	85 (40.4)	2 (7.4)	Reference	0.002		
Sepsis	43 (20.5)	11 (40.8)	10.87 (2.74–72.30)			
Ischemic	49 (23.3)	10 (37.0)	8.67 (2.17–57.93)			
Nephrotoxicity	31 (14.8)	3 (11.1)	4.11 (0.65–32.34)			
Glomerulonephritis	2 (1.0)	1 (3.7)	21.25 (0.80–358.95)			
Cause of liver disease, *n* (%)						
Alcohol‐related	29/210 (13.8)	3/27 (11.1)	Reference	0.0181		
Autoimmune	11/210 (5.2)	0/27 (0)				
Biliary	11/210 (5.2)	2/27 (7.4)	1.76 (0.21–12.05)			
Hepatitis	78/210 (37.1)	15/27 (55.5)	1.86 (0.56–8.44)			
Hereditary	11/210 (5.2)	0/27 (0)				
SALF	30/210 (14.3)	0/27 (0)				
Other	40/210 (19.0)	7/27 (25.9)	1.69 (0.43–8.36)			
BMI (median, IQR)	26.3 (23.4–30.1)	27.3 (24.7–30.9)	1.03 (0.94–1.12)	0.5197		
MELD (median,dp)						
(median, IQR)	30 (10)					
30 (24–36)	31.8 (8.4)					
32 (25–40)	1.02 (0.98–1.06)	0.3802				
Era, *n* (%)^∗^						
Pre‐MELD	35/210 (16.7)	5/27 (18.6)	Reference	0.886		
Post‐MELD Period 1	79/210 (37.6)	11/27 (40.7)	0.97 (0.32–3.28)			
Post‐MELD Period 2	96/210 (45.7)	11/27 (40.7)	0.80 (0.27–2.69)			
Donor type, *n* (%)						
Living	11/210 (5.2)	0/27 (0)				
Deceased	198/210 (94.3)	27/27 (100%)				
Domino transplant	1 (0.5)	0/27 (0)				
Initial dialysis modality, *n* (%)						
CVVHDF	119/210 (56.7)	14/27 (51.8)	Reference	0.6359		
Intermittent HD	91/210 (43.3)	13/27 (48.1)	1.21 (0.54–2.72)			
Graft dysfunction, *n* (%)	50/210 (23.8)	10/27 (37.0)	1.88 (0.79–4.32)	0.1511		
Type of dysfunction, *n* (%)						
Early	37/210 (17.6)	6/27 (22.2)	Reference	0.3824		
Late	13/210 (6.2)	4/27 (14.8)	1.90 (0.43–7.76)			
Retransplantation, *n* (%)	47/210 (22.4)	5/27 (18.5)	0.79 (0.25–2.05)	0.6422		
Sepsis, *n* (%)	31/210 (14.7)	5/27 (18.5)	1.31 (0.42–3.49)	0.6172		
Immunosuppressive drug, *n* (%)						
Tacrolimus	198/210 (94.3)	27/27 (100)				
Cyclosporine	12/210 (5.7)	0/27 (0)				
Timing of dialysis, *n* (%)						
Preliver transplantation	62/210 (29.5)	12/27 (44.4)	Reference	0.1249		
Postliver transplantation	148/210 (70.5)	15/27 (55.5)	0.52 (0.23–1.20)			
ICU LOS (days) (median, IQR)	11 (6–17.8)	16 (8–39.5)	1.04 (1.02–1.06)	< 0.001		
Hospital LOS (days) (median, IQR)	34.5 (24–50.8)	40 (24–88.5)	1.02 (1.01–1.04)	< 0.001		

*Note:* Results presented as median (interquartile range) or n/total (%). OR, odds ratio; 95% CI, 95% confidence interval; CVVHDF, continuous venovenous hemodiafiltration; HD, hemodialysis. Because eGFR incorporates age, sex, and baseline creatinine, it was selected over individual variables to avoid multicollinearity. All VIF values were below 1.008, indicating no multicollinearity, and the assumption of linearity for eGFR was met. The Hosmer–Lemeshow test showed good model calibration (*X* ^2^ = 5.27; *p* = 0.73).

Abbreviaitons: BMI, body mass index; GFR, glomerular filtration rate; LOS, length of stay; MELD, Model for End‐Stage Liver Disease; SALF, severe acute liver failure.

^∗^Era: pre‐MELD (January 1, 1999 to June 30, 2006), post‐MELD Period 1 (July 1, 2006 to December 31, 2010), and post‐MELD Period 2 (January 1, 2011 to December 31, 2015).

^#^Data available for 210/228 patients (92.1%).

### 3.4. Liver Graft Survival at 90 Days Among Liver Transplant Recipients Who Developed an Episode of AKI‐D

Among the 356 patients included in the study, 89 underwent retransplantation and 267 received a single transplant. Eight retransplanted patients had undergone their primary transplantation at another institution several years earlier and were excluded from the graft survival analysis due to the inability to determine the onset of graft dysfunction. Thus, 81 retransplanted patients were analyzed: 6 received 3 grafts (2 retransplantations), and 75 received 2 grafts (1 retransplantation), totaling 435 grafts included in the analysis.

At 90 days posttransplantation, the estimated probability of graft survival was 81.0% (95% CI: 0.77–0.85). The risk of graft failure was twice as high in patients who initiated dialysis after transplantation compared to those who initiated it before transplantation (Figure 2) (Model 1: HR = 2.10, 95% CI: 1.11–4.00, *p* = 0.014; Model 2: HR = 2.32, 95% CI: 1.22–4.42, *p* = 0.005) (Table 4).

**FIGURE 2:**
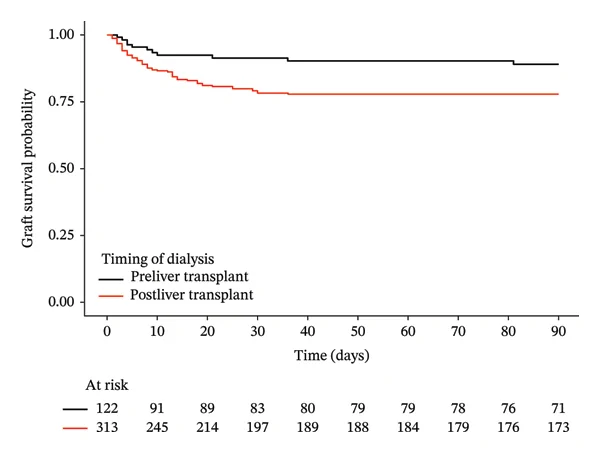
Graft survival according to the timing of dialysis initiation (*n* = 435 grafts).

**TABLE 4:** Univariable and multivariable Cox regression analyses of factors associated with 90‐day graft survival among liver transplant recipients receiving dialysis before or after transplantation.

Predictors	N 435 (100%)	Univariate analysis	*p* value	Multivariate analysis Model 1	*p* value	Multivariate analysis Model 2^∗∗^	*p* value
		HR (95% CI)		HR (95% CI)		HR (95% CI)	
Age, *n* (%)							
< 65 years	379/435 (87.1)	Reference	0.5761				
≥ 65 years	56/435 (12.9)	0.81 (0.39–1.69)					
Sex, *n* (%)							
Female	182/435 (41.8)	Reference					
Male	253/435 (58.2)	0.76 (0.48–1.20)	0.2427				
Comorbidities, *n* (%)							
Systemic arterial hypertension	34/435 (7.8)	0.80 (0.32–1.98)	0.6296				
Diabetes mellitus	19/435 (4.4)	0.52 (0.13–2.12)	0.3545				
Heart disease	6/435 (1.4)	0.86 (0.12–6.19)	0.8838				
Cause of liver disease, *n* (%)							
Alcohol‐related	49/435 (11.3)	Reference	0.4006				
Autoimmune	21/435 (4.8)	1.17 (0.29–4.67)					
Biliary	19/435 (4.4)	0.37 (0.04–3.09)					
Hepatitis	176/435 (40.5)	1.58 (0.66–3.78)					
Hereditary	23/435 (5.3)	2.61 (0.84–8.09)					
SALF	52/435 (11.9)	1.23 (0.41–3.67)					
Other	95/435 (21.8)	1.75 (0.70–4.37)					
Initial dialysis modality, *n* (%)							
Intermittent HD	150/435 (34.5)	Reference	0.0458	Reference	0.0225	Reference	0.0198
CVVHDF	285/435 (65.5)	1.68 (1.00–2.80)		1.78 (1.06–2.98)		1.81 (1.08–3.04)	
SALF, *n* (%)	52/435 (11.9)	0.82 (0.38–1.79)	0.6260				
Donor type, (*n*%)							
Deceased	392/435 (90.1)	Reference	0.0569				
Living	43/435 (9.9)	1.85 (0.97–3.51)					
Sepsis, *n* (%)	67/435 (15.4)	0.59 (0.27–1.29)	0.1845				
Timing of dialysis, *n* (%)							
Preliver transplant	122/435 (28.0)	Reference	0.0132	Reference	0.014	Reference	0.005
Postliver transplant	313/435 (72.0)	2.20 (1.16–4.18)		2.10 (1.11–4.00)		2.32 (1.22–4.42)	
Baseline eGFR, (mL/min/1,73 *m* ^2^), *n* (%)							
≥ 60	374/435 (86.0)	Reference	0.0172	Reference	0.0053		
30–59	42/435 (9.6)	0.31 (0.10–0.99)		0.32 (0.10–1.03)			
< 30	19/435 (4.4)	0^•^		0^•^			
MELD^∗^ score, points, *n* (%)							
0–20	88/422 (20.9)	Reference	0.1462				
21–30	142/422 (33.6)	0.67 (0.37–1.20)					
31–40	140/422 (33.2)	0.55 (0.30–1.02)					
> 40	52/422 (12.3)	0.44 (0.18–1.08)					

*Note:* Results presented as n/total (%). HR, hazard ratio; 95% CI, 95% confidence interval; CVVHDF, continuous venovenous hemodiafiltration; HD, hemodialysis. For both multivariable models, the global test for proportional hazards confirmed that the assumption was met (*X* ^2^ = 2.34, *p* = 0.67 for Model 1; *X* ^2^ = 1.64, *p* = 0.44 for Model 2). No graft failure occurred within 90 days in this group.

Abbreviations: GFR, glomerular filtration rate; MELD, Model for End‐Stage Liver Disease; SALF, severe acute liver failure.

^∗^The MELD score was available for 422 out of 435 liver grafts (97.0%).

^#^Model 1: Multivariable model based on a set of variables including baseline eGFR.

^∗∗^Model 2: Multivariable model based on a set of variables including MELD score.

## 4. Discussion

In our study, the timing of dialysis initiation (pre‐ vs. postliver transplantation) was not associated with patient survival or dialysis dependence at 90 days. The majority of surviving patients reverted to conservative nephrological management by the 90‐day follow‐up. However, posttransplant dialysis initiation correlated with a higher risk of liver graft dysfunction.

AKI is a critical complication for liver transplant candidates and recipients [5, 6]. In liver transplantation, it is associated with increased morbidity, mortality, reduced graft survival, prolonged hospital stays, and higher healthcare costs [27–29, 45]. Since the implementation of the MELD score in 2002 [38], there has been a marked increase in the proportion of transplant candidates and recipients presenting with renal dysfunction [10], alongside a corresponding rise in the frequency of simultaneous liver–kidney transplants [32].

Predicting which patients with renal dysfunction will progress to end‐stage kidney disease or require a kidney allograft following liver transplantation remains a significant challenge [32]. In 2017, the United Network for Organ Sharing (UNOS)/Organ Procurement and Transplant Network (OPTN) implemented a “safety net” policy to ensure the rational allocation of kidney grafts and to guarantee that liver transplant recipients who developed advanced renal disease have the opportunity to receive a kidney transplant with priority status between 60 and 365 days postliver transplantation [46]. Given that dialysis forms part of supportive therapy for patients awaiting liver transplantation [8], we included patients with liver cirrhosis, ACLF, and SALF in our analysis.

We focused on patients with KDIGO Stage 3 dialysis–requiring AKI to capture the group most vulnerable to poor outcomes. We limited the time interval to 4 weeks before and after transplantation to confirm the acute nature of dialysis support, avoiding overlap with the 6‐week period required for simultaneous liver–kidney transplant evaluation [46]. Our objective was to assess whether the timing of dialysis initiation impacted mortality and short‐term outcomes in liver transplant recipients who developed an episode of AKI‐D.

In our cohort, the choice of dialysis initiation, dialysis prescription, as well as transitions between modalities was at the discretion of the care team. Nephrological assessment was managed by a dedicated group of nephrologists, several of whom were involved in patient care throughout the whole study period. While equipment and protocols naturally evolved over time, based on new scientific evidence and materials available, there was uniformity in the quality of renal support over the 16‐year period. Patients undergoing dialysis before versus after liver transplantation differed in several respects; however, no significant differences were observed in hospital LOS or duration of dialysis therapy between the groups.

In our study, the etiological classification of AKI was based primarily on clinical criteria rather than histological diagnosis. As suggested by Davenport et al. [47], our classification represented injury phenotypes in which specific pathophysiological mechanisms were more prominent. HRS and sepsis‐associated AKI were the predominant mechanisms for AKI in our cohort. However, limitations inherent to the multifactorial etiology may introduce inaccuracies into the clinical classification. Even in cirrhotic patients with ascites—where HRS represents a distinct renal dysfunction phenotype—other nephropathies may coexist [48]. The concept that HRS is a functional renal injury has been challenged, as some degree of parenchymal changes may be present [7, 49]. Infectious complications represent a critical trigger for liver disease decompensation [48, 50, 51] and can overlap with other lesions. Coagulopathy often precludes histological confirmation of nephropathy in patients with severe hepatic failure. Only three patients were diagnosed with glomerulonephritis based on kidney biopsy findings (IgA nephropathy, cryoglobulinemia, and focal segmental glomerulosclerosis). We consider that diagnostic challenges and the small number of patients with glomerulopathy limit the interpretation of the impact of AKI etiology on the evaluated outcomes. Furthermore, although reversible by transplantation, HRS was not an independent risk factor for 90‐day mortality when compared to other causes of AKI [52].

Consistent with findings by Wong et al. [8], continuous KRT was the most frequently employed initial modality in liver transplant patients and was associated with higher mortality. Since dialysis modality selection depends on clinical condition—with continuous KRT reserved for the most critically ill—this association likely reflects disease severity rather than being an independent risk factor, as similarly suggested by Gonwa [53] and Cama‐Olivares et al. [52].

The 90‐day mortality rate in our sample was 28.4%, comparable to the 28.6% reported by Gonwa et al. between 1985 and 1995, and lower than the mortality rates reported by the same author during 1996–1999 (39.7%) [53] and by Arani et al. (44%) in a more recent study [26]. When analyzing the timing of dialysis initiation—pre‐ versus posttransplant—no significant difference in 90‐day mortality was observed. Wong et al. [8] similarly reported comparable mortality rates among patients starting dialysis before or after liver transplantation. Clinical severity at dialysis initiation and the success of liver transplantation emerged as the two most critical determinants of short‐term survival.

Renal failure adversely impacts both patient and liver graft survival [42, 54]. AKI appears to be bidirectionally linked to liver graft dysfunction. Cabezuelo et al. and Wadei et al. [28, 29] identified graft dysfunction as a risk factor for posttransplant AKI, while Hilmi et al. [14] demonstrated that AKI within the first 72 h posttransplant significantly reduces graft survival. Thongprayoon et al. [19] also reported increased graft dysfunction risk in patients with dialysis‐requiring AKI following liver transplantation. AKI after transplantation is thus both a localized renal pathology and a marker of multiorgan injury [20]. Our finding of a higher incidence of severe graft dysfunction and increased retransplant rates in patients who initiated dialysis postoperatively further supports the association between posttransplant AKI and reduced graft survival. Among the 356 patients analyzed, 25% required retransplant, with those starting dialysis after transplantation exhibiting a significantly higher risk of graft failure.

Similar to other clinical settings, AKI in liver transplantation is a recognized risk factor for CKD. The severity, duration, and recurrence of AKI episodes contribute to poorer renal outcomes, including progression to CKD and dialysis dependence [29, 54]. In our cohort, the timing of dialysis initiation, pre‐ or posttransplant, did not influence dialysis dependence at 90 days. Predictors of persistent renal dysfunction included a history of diabetes mellitus and pretransplant kidney disease, suggesting that impaired physiological reserve plays a role in renal prognosis.

Despite these risks, most liver transplant recipients recover renal function and discontinue dialysis during early follow‐up. Even patients with CKD may experience renal recovery posttransplant; Hilmi et al. [14] reported that over 60% of patients with Stage 4 and Stage 5 CKD recovered renal function within the first year after transplantation. In our sample, only 11% of survivors remained dialysis‐dependent at 90 days. Notably, although SALF is associated with a high incidence of acute renal failure and dialysis need in approximately 30% of cases, it seldom leads to CKD [55]. None of the SALF patients in our cohort required ongoing renal support at 3 months posttransplant.

Our study has several limitations. First, due to its observational nature, causality cannot be established; the findings can only generate hypotheses. Second, as a single‐center study based on patients treated several years ago, our findings may reflect specific institutional practices or changes in clinical and surgical protocols over time. Reflecting a global trend underway since the early 2000s, the Enhanced Recovery After Surgery (ERAS) protocol fostered the adoption of measures aimed at reducing perioperative stress and improving short‐term outcomes after major surgery [56, 57]. Over the past 2 decades, several measures have been incorporated into the care of liver transplant patients at our institution. The piggyback technique, which avoids venovenous bypass during liver transplantation, became the predominant surgical approach. Viscoelastic tests became preferred for coagulation assessment and helped guide the transfusion of blood derivatives and blood components in patients with liver disease. Hemodynamic monitoring and volume status assessment contributed to minimizing the occurrence of hypervolemia and its consequences. Defining a target mean arterial pressure greater than 60–65 mmHg contributed to establishing more rational hemodynamic goals in patients with liver disease. Ventilatory adjustments aimed at preventing hyperoxia and ensuring protective ventilation became widespread in both the ICU and the operating room. Multimodal analgesia contributed to reducing opioid use and, consequently, its undesirable side effects. Encouraging extubation, mobilization, device removal, and the earliest possible initiation of nutritional support became standard protocol [58]. The use of hydroxyethyl starch 130/0.42 was banned due to its association with unfavorable outcomes [59]. However, this set of measures was integrated into clinical practice gradually, with considerable overlap, making it impossible to establish precise dates for each specific intervention adoption in the institution. In an attempt to encompass the implementation of the MELD score and different periods in the intensive care of liver transplant patients, we analyzed the impact of three distinct periods (pre‐MELD: 1999 to June 2006; post‐MELD Period 1: July 2006 to 2010; and post‐MELD Period 2: 2011–2015). Although the periods were associated with the timing of dialysis initiation, they did not correlate with higher mortality or dependence on renal replacement therapy. Conceivably, changes in care protocols constituted a continuum throughout the study period, blurring the differences among the three periods.

Third, because our analysis focused on care provided in the ICU, we lack complete donor data and detailed intraoperative information, such as hemorrhagic complications, the need for and extent of transfusion support, the occurrence of postreperfusion syndrome, and other risk factors associated with posttransplant renal dysfunction. Fourth, our cohort was limited to ICU‐admitted patients, restricting our analysis to the most critically ill individuals who are inherently at higher risk for adverse outcomes.

Finally, the 90‐day follow‐up period prevents us from evaluating the long‐term impact of dialysis‐requiring AKI and from developing strategies for the nephrological follow‐up of these patients during extended posttransplant care.

## 5. Conclusion

AKI is a common complication in both the pre‐ and postliver transplantation periods and is associated with increased morbidity and mortality. Dialysis is a component of supportive therapy for patients awaiting transplantation as well as for liver graft recipients. In our study, the timing of dialysis initiation—pre‐ versus postliver transplantation—had no impact on patient survival or dialysis dependence at 90 days. However, initiation of dialysis after transplantation was associated with a higher risk of liver graft dysfunction.

**Nomenclature**
ACLF: Acute‐on‐chronic liver failure
AKI: Acute kidney injury
AKI‐D: KDIGO Stage 3 dialysis–requiring acute kidney injury
CKD: Chronic kidney disease
CVVHDF: Continuous venovenous hemodiafiltration
DAMPs: Damage‐associated molecular patterns
GFR: Glomerular filtration rate
HRS: Hepatorenal syndrome
ICU: Intensive care unit
IHD: Intermittent hemodialysis
KDIGO: Kidney Disease: Improving Global Outcomes
KRT: Kidney replacement therapy
MELD: Model for End‐Stage Liver Disease
PAMPs: Pathogen‐associated molecular patterns
SALF: Severe acute liver failure

## Author Contributions

Conceptualization: P.F.S. and M.S.D.J. Data curation: P.F.S., M.S.D.J., and T.D.C. Formal analysis: P.F.S. and M.S.D.J. Investigation: P.F.S., A.L.A., B.F.C.S., C.D.B., J.C.M.M., M.C.B., M.P.D., O.F.P.S., T.N.M., T.D.C., V.G.P.J., and M.S.D.J. Methodology: P.F.S. and M.S.D.J. Project administration: P.F.S. Supervision: M.S.D.J. Validation: P.F.S., M.S.D.J., and T.D.C. Writing–original draft: P.F.S. Writing–review anda editing: M.S.D.J., T.D.C., A.L.A., B.F.C.S., J.C.M.M., M.C.B., M.P.D., O.F.P.S., T.N.M., and V.G.P.J.

## Funding

The authors received no specific funding for this work.

## Ethics Statement

This study was approved by the Research Ethics Committee (CEP) of the Federal University of São Paulo, with a waiver of informed consent. Informed consent was waived due to the retrospective nature of the study and because anonymized data were used. We declare that our research was conducted on human data in compliance with the Helsinki Declaration (https://www.wma.net/policies-post/wma-declaration-of-helsinki/).

## Consent

Please see the Ethics Statement.

## Conflicts of Interest

The authors declare no conflicts of interest.

## Data Availability

The datasets analyzed during the current study are currently under review by the curation team of the Dryad repository (digital object identifier: https://doi.org/10.5061/dryad.j9kd51ctf).
